# Microalgae supplementation improves goat milk composition and fatty acid profile: a meta-analysis and meta-regression

**DOI:** 10.5194/aab-68-223-2025

**Published:** 2025-03-26

**Authors:** Soumaya Boukrouh, Ihssane Mnaouer, Poliana Mendes de Souza, Jean-Luc Hornick, Abdelaziz Nilahyane, Bouchra El Amiri, Abdelaziz Hirich

**Affiliations:** 1 African Sustainable Agriculture Research Institute (ASARI), Mohammed VI Polytechnic University (UM6P), Laâyoune 70000, Morocco; 2 Animal Production Unit, Regional Center Agricultural Research of Settat, National Institute for Agricultural Research (INRA), Rabat 10090, Morocco; 3 Department of Veterinary Management of Animal Resources, Faculty of Veterinary Medicine, University of Liège, 4000 Liège, Belgium

## Abstract

Recently, there has been an emphasis on research on sustainable and environmentally friendly agricultural practices. Microalgae are a promising feed that is rich in essential nutrients, and research has been oriented toward their incorporation into ruminant diets. This study aimed to evaluate the inclusion of microalgae in goat diets using a meta-analysis methodology. The data were acquired from 17 peer-reviewed scientific papers. The raw mean difference between the treatment diets supplemented with microalgae and the control diets was evaluated using the random-effects model. Experimental characteristics such as animal breed, days in milk, experimental duration, microalgae species, inclusion levels, and concentrate were used as covariates in meta-regression and subgrouping analyses. Microalgae supplementation did not affect dry matter intake (DMI, p=0.170 ) but significantly improved the intake of crude protein (CP, p<0.001 ) and neutral detergent fiber (NDF, p=0.005 ). The incorporation of microalgae into the goat diet improved all digestibility parameters ( p<0.01 ), with an improvement in fermentation parameters, including ruminal pH ( p=0.010 ) and propionate ( p<0.001 ). Microalgae inclusion in goat diets increased blood glucose levels ( p<0.001 ) but did not affect blood antioxidant activity ( p>0.05 ). Microalgae supplementation did not affect milk yield ( p=0.480 ) but increased the yield of lactose ( p<0.001 ), protein ( p<0.001 ), and fat ( p<0.001 ). Microalgae inclusion in goat diets improves the fatty acid (FA) profile. The milk of goats had significantly decreased C18:0 ( p=0.001 ) and C18:1 n-9 ( p=0.028 ) and increased C20:5 n-3 ( p=0.027 ), C22:6 n-3 (DHA, p<0.001 ), polyunsaturated FA (PUFA, p=0.039 ), and n-3 ( p=0.006 ). Subgroup analysis showed that an inclusion level higher than 30 g per kg DM was advisable to obtain this total improvement. However, even a moderate inclusion level (15–30 g per kg DM) improved the FA profile. The microalgae species *Schizochytrium* sp. and the Alpine–Greek crossbreed goat breed were covariates that showed interesting results concerning the improvement of DHA and PUFA. In addition, supplementation of goat diets with microalgae could be used as a nutritional approach to enhance milk production and quality.

## 1 Introduction

Ruminant milk is one of the most popular beverages in the world. It is a substantial contributor to global food security as an important source of protein, fat, sugar, minerals, and vitamins (Moore et al., 2023). In fact, the world population is envisaged to grow, and inquiries into animal products will increase in parallel (Smith et al., 2022). In Morocco, goat milk production reached 46 227 t in 2018 (FAOSTAT, 2022). Moreover, in rural and dry areas, camel and goat breeding is a fundamental economic activity that contributes to breeder income and cultural heritage (Ait El Alia et al., 2025; Boukrouh et al., 2023a, 2024c).

However, livestock farming increases the pollution from natural resources (Sakadevan and Nguyen, 2017). Intensive livestock farming contributes significantly to air pollution, with the release of greenhouse gases including carbon dioxide methane and nitrous oxide, significantly accelerating climate change (Sakadevan and Nguyen, 2017). Livestock production is associated with nutrient loss, soil degradation, and grassland conservation (Boukrouh et al., 2024a). In this context, research on alternative feedstuffs as a substitute for standard feedstuffs has recently increased (Boukrouh et al., 2023b, c; 2024b; Hirich et al., 2021, 2020), especially in terms of overcoming some environmental problems related to salinity or when the costs of traditionally used feedstuffs are very high.

Microalgae are microscopic photosynthetic organisms capable of transforming sunlight and carbon dioxide (CO_2_) into valuable biomass. Recently, microalgae cultivation has been developed and used as an unconventional animal feed owing to its environmental and economic advantages (Khan et al., 2018). Algae are highly efficient in converting solar energy, which gives them a rapid growth rate and increases their production compared with traditional crops (Khan et al., 2018). Moreover, microalgae do not rely on external environmental factors. They can substantially valorize water that is unsuitable for humans, thus reducing the pressure on cultivated lands (Holman and Malau-Aduli, 2013).

Microalgae possess abundant essential and healthy unsaturated fatty acids (UFAs), docosahexaenoic acid (DHA), and eicosapentaenoic acid (EPA), which are present in lower amounts in ruminant feeds (Kholif and Olafadehan, 2022). Their composition varies according to the algal growth conditions, species, and genus (Kholif and Olafadehan, 2022). The green freshwater microalgae *Chlorella vulgaris*, belonging to the family Chlorellaceae and the class Trebouxiophyceae, is a primary source of linoleic (C18:2 n-6) and α -linolenic (C18:3 n-3) beneficial FA (Pantami et al., 2020). The microalgae *Japonochytrium* sp. is a saprophytic species belonging to the kingdom Thraustochytrid, which produces and accumulates high amounts of DHA in its biomass (Jaseera and Kaladharan, 2020). *Schizochytrium* sp. microalgae are rich in nutrients, such as sugar and protein, and contain a higher fat content (18.3 % DM–25 % DM) than other microalgae. The FA profile of *Schizochytrium* sp. is rich in UFA and is composed of palmitic acid (C16:0), docosapentaenoic acid (DPA), and DHA (Zhu et al., 2022). These microalgae UFAs can contribute to improving ruminal fermentation and feed digestibility (Sofyan et al., 2022) and thus to improving the transfer of UFA or conjugated linoleic acid (CLA) to animal products. CLA has anti-atherogenic, anti-carcinogenic, and anti-obesity properties (Vignaud et al., 2023). Microalgae are also a major source of proteins, essential amino acids, and other health-promoting nutrients, making them attractive feed supplements for livestock production (Mavrommatis and Tsiplakou, 2020).

A meta-analysis evaluating microalgae incorporation into goat kid, poultry, and pig diets showed interesting results and performance improvement (Martins et al., 2021; Orzuna-orzuna et al., 2021). Nevertheless, studies on milk production and FA profiles of ruminants incorporating microalgae are still scarce (Martins et al., 2021). Notably, some studies have evaluated the effects of incorporating microalgae into lactating-goat diets on milk production, chemical composition, FA, and fermentation profile. However, the results of these studies are controversial and inconclusive. For example, the incorporation of low doses (5 g) of microalgae into a goat diet increased milk production, chemical composition, FA profile, and fermentation parameters (Kholif et al., 2022). Conversely, the incorporation of low doses in other studies had no significant effect on milk yield and quality (Mavrommatis et al., 2018; Tsiplakou et al., 2018). According to Martins et al. (2021), previous studies have provided valuable insights, but the body of literature remains diverse, encompassing variations in microalgae species, doses of inclusion, animal breeds, and periods of supplementation.

A few recently published review articles have demonstrated the importance of microalgae inclusion in ruminant diets for the improvement of milk production and quality in ruminants (Altomonte et al., 2018; Hernández et al., 2022). However, these studies did not apply meta-analytic methods to assess the effects of microalgae on goats. According to Viechtbauer (2010), meta-analysis is a statistical approach offering unbiased evidence regarding the effectiveness of a treatment by aggregating and quantitatively analyzing data from various previously published studies. The hypothesis of the present meta-analysis asserts that the incorporation of microalgae into lactating-goat diets will ameliorate goat milk production, chemical composition, and FA profile. Consequently, the objective of this study was to evaluate, through a meta-analytic procedure, the impact of dietary supplementation with microalgae on animal performance and milk production and quality.

## 2 Materials and methods

### 2.1 Literature search

**Figure 1:**
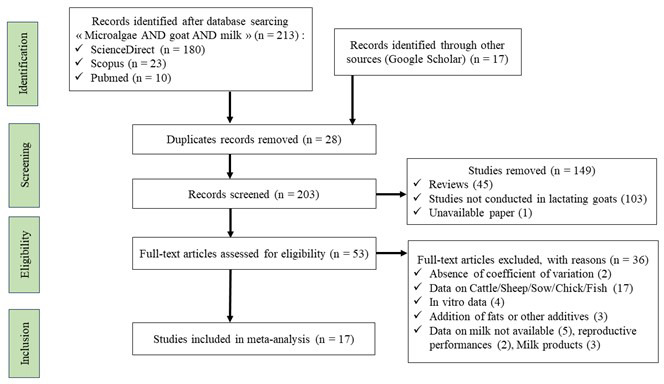
Flowchart of article selection based on the PRISMA protocol.

A systematic literature search was performed using Science Direct, Scopus, PubMed, and Google Scholar to identify scientific papers that evaluated the effects of microalgae in goat diets. Google Scholar searches were performed by browsing the articles until the results were repeated. The database search was performed thrice. The search approach of the systematic review was based on PICO: population, intervention, comparator, and outcome (Schiavenato and Chu, 2021). The following terms were used for the population: goat or dairy goat. The intervention concerned microalgae in goat diets. The comparator was viewed as an animal receiving the same treatment, with or without microalgae supplementation. The outcome was milk production and quality (milk or lactation). The studies used in the meta-analysis spanned a period of 7 years, starting from the first publication in 2015 to the most recent publication in 2022. The identification, selection, and inclusion of studies adhered to the guidelines outlined in the Preferred Reporting Items for Systematic Reviews and Meta-Analyses (PRISMA) protocol (Al Gharad et al., 2025) (Fig. 1).

The requirements for papers to be included in the meta-analysis were as follows: (i) the papers must be peer-reviewed original research papers published in English that report on, at least, the available lactational performance data in goats, with the data mentioning the standard error (SE) or standard deviation (SD); (ii) the study must have both control and experimental treatments that were fed microalgae; (iii) the experimental diets were free from microalgae protection by fat or any other growth promoters; and (iv) the goats used in the feeding trials were healthy and clinically safe. In total, 17 studies were considered to be suitable for meta-analysis, and a description of the experimental conditions is shown in Table 1.

### 2.2 Data extraction

Papers meeting the inclusion criteria were rated by publication reference, country, number of animals, animal breed, days in milk, experimental period, microalgae species, inclusion level, and amount of concentrates in the date (g per kg DM). Variables reported in three or more publications were the only variables considered. Data were excluded if duplicates were found in more than one journal. The following parameters were included in the final database: intake of dry matter (DMI), organic matter (OMI), crude protein (CPI), and neutral detergent fiber (NDFI); digestibility of DM (DMD), OM (OMD), CP (CPD), NDF (NDFD), ADF (ADFD), and ether extract (EED); fermentation parameters (ruminal pH, NH_3_, acetate, propionate); blood parameters (total protein, albumin, globulin, glucose); antioxidant activity (superoxide dismutase (SOD), catalase (CAT), glutathione reductase (GR), glutathione transferase (G-Tr), and glutathione peroxidase (G-Px)); milk yield and chemical composition (total solids, ash, fat, protein, solids not-fat, protein, lactose, fat, solids-not fat); and FA profile (stearic (C18:0), oleic (C18:1 n-9), linoleic (C18:2 n-6), conjugated linoleic (C18:2 c9t11), α -linolenic (C18:3 n-3), arachidonic (C20:4 n-3), eicosapentaenoic (C20:5 n-3), docosahexaenoic (C22:6 n-3)) and FA ratios and summaries (saturated FA (SFA), monounsaturated FA (MUFA), polyunsaturated FA (PUFA), n-3, n-6, C14:1 : C14:0, C16:1 : C16:0, and atherogenicity index).

**Table 1:** Summary of the studies included in the meta-analysis.

Reference	Country	n	Goat breed	Days	Exp.	Microalgae species	Inclusion	CON
		animals		in	period		level (g per	(g per
				milk	(d)		kg DM)	kg DM)
Fougère et al. (2018)	France	12	Alpine	86	28	*Schizochytrium* sp.	20–60	550
Kholif et al. (2017)	Egypt	15	Damascus	7	84	*Chlorella vulgaris*	5–10	500
Kholif et al. (2020a)	Egypt	32	Boer	7	84	*Chlorella vulgaris*	10	500
Kholif et al. (2020b)	Egypt	15	Nubian	7	28	*Nannochloropsis oculata*	5–10	500
Kholif et al. (2022)	Egypt	15	Damascus	7	30	*Chlorella vulgaris*	10	400
Marques et al. (2021)	Brazil	38	Anglo-Nubian	NR	35	*Chlorella pyrenoidosa*	<15	NR
			crossbreed					
Mavrommatis et al. (2018)	Greece	24	Alpine–local Greek	150	74	*Schizochytrium* sp.	20–60	500
Mavrommatis and	Greece	24	Alpine–local Greek	150	74	*Schizochytrium* sp.	20–60	500
Tsiplakou (2020)								
Mavrommatis et al. (2021)	Greece	24	Alpine–local Greek	150	74	*Schizochytrium* sp.	20–60	500
Martin et al. (2021)	France	4	Alpine	NR	28	*Schizochytrium* sp.	39	NR
Novotná et al. (2017)	Czech Republic	45	White Short-Haired	NR	102	*Chlorella vulgaris* and *Japonochytrium* sp.	10	NR
Pajor et al. (2019)	Hungary	40	Native Hungarian	71	31	*Schizochytrium limacinum*	15	285.7
Pajor et al. (2021)	Hungary	28	Alpine	161	35	*Schizochytrium limacinum*	10	285.7
Póti et al. (2015)	Hungary	20	Native Hungarian	62	17	*Chlorella kessleri* and *Spirulina platensis*	10	331
Tsiplakou et al. (2018)	Greece	16	Crossbreed	94	28	*Chlorella vulgaris*	5	470
Tsiplakou et al. (2017)	Greece	16	Crossbreed	94	28	*Chlorella pyrenoidosa*	5	500
Zhu et al. (2022)	China	120	Guanzhong	NR	65	*Schizochytrium* sp.	15–35	NR

n denotes number, exp. denotes experimental, and CON denotes concentrate in the diet. NR denotes not reported.

The number of repetitions, means, and variances (i.e., SE and SD) were extracted for the control (without microalgae addition) and treatments (with microalgae addition). In studies that reported SE instead of SD, the SE values were converted to SD using the following formula: SD = SE × $\sqrt{n}$ , where n is the number of goats in each treatment group (Xue et al., 2019). Also, when a study reported the incorporation level in terms of percentage (%) instead of g kg^−1^, the percentage value was converted to g kg^−1^ using the following formula: 1 % = 10 g kg^−1^. In feeding assays with variable treatments, data were only extracted from the microalgae and control groups, irrespective of the other treatments.

### 2.3 Statistical analysis

Statistical analysis of the collected data was performed using a meta-analysis. Data were analyzed using the “meta” and “metafor” packages of R software (Version 4.4.1). All outcomes in subsequent studies were continuous data. The raw mean difference (RMD) with a 95 % confidence interval (CI) was selected for the calculation and combination of effect measures. The significance of the combined effect sizes was evaluated using the Z test, and treatment had a distinct effect when p≤0.05 .

Publication bias was detected using Egger's test (1997), and the bias was considered to be present when p≤0.05 . Heterogeneity was quantified using chi-squared ( Q ) and $I^{2}$ tests (Riley et al., 2011). In a meta-analysis, $I^{2}$ indicates the percentage of variance assigned to study heterogeneity. $I^{2}$ ranges from 0 % to 100 % and is considered to be present when it exceeds 50 % and when p≤0.05 (Langan, 2022).

Meta-regression was used to investigate potential contributors to heterogeneity. The meta-regression criteria for the heterogeneity test and for Egger's test was p≤0.05 . Meta-regression analysis were performed using the subsequent covariates: (1) animal breed (native Hungarian, White Short-Haired, Alpine–Greek crossbreed, Nubian, crossbreed, Guanzhong, Boer, Alpine, Damascus, Anglo-Nubian crossbreed), days in milk ( <90 , 90–180 d), experimental period ( <90 , >90 d), microalgae species (*Chlorella vulgaris, Chlorella pyrenoidosa, Japonochytrium* sp., *Nannochloropsis oculata, Schizochytrium* sp., *Schizochytrium limacinum, Chlorella kessleri* and *Spirulina platensis*), microalgae inclusion level ( <10 , 11–20, 21–30, 31–40, 41–50, 51–60 g per kg DM), and the level of concentrate in the diet ( <400 , 400–600 g per kg DM).

Subgroup analysis was also used to elucidate heterogeneity when the covariates (from the meta-regression procedure in Table 4) were significant at p≤0.10 . The subgroup assessment was conducted for variables reported in at least 10 studies in the meta-analysis. The covariate was compelling with regard to explaining the heterogeneity when the p value for the subgroup differences was less than 0.05 (Table 4).

## 3 Results

The database comprises 17 peer-reviewed scientific papers with 29 chosen treatment means to explore the effects of microalgae incorporation into dairy goat diets on their performance and milk quality (Table 1). A total of 953 dairy goats, comprising 1061 comparisons, were used in the meta-analysis.

### 3.1 Intake, digestibility, and fermentation parameters

The results revealed that there was no evidence of an intervention effect on dry matter intake (DMI, p=0.170 ) and organic matter intake (OMI, p=0.227 ). However, a significant difference was found in crude protein intake (CPI, p<0.001 ) and neutral detergent fiber intake (NDFI, p=0.005 ) (Table 2). Microalgae supplementation in goat diets significantly improved dry matter digestibility (DMD, p=0.001 ), organic matter digestibility (OMD, p<0.001 ), crude protein digestibility (CPD, p=0.001 ), neutral detergent fiber digestibility (NDFD, p=0.012 ), acid detergent fiber digestibility (ADFD, p<0.001 ), and ether extract digestibility (EED, p=0.003 ). Microalgae supplementation significantly increased the ruminal pH ( p=0.010 ) and propionate ( p<0.001 ). Heterogeneity was discovered in all previous parameters ( p<0.05 , $61.56<I^{2}<96.22$ ), except in ADFD ( p=0.492 , $I^{2}=0$ ) and propionate ( p=0.295 , $I^{2}=0$ ).

**Table 2:** Effects of microalgae supplementation on feed intake, nutrient digestibility, fermentation, and blood parameters of dairy goat diets.

	Control mean (SD)	N	RMD (95 % CI)		Heterogeneity		Bias
			Random effect	p value	p value	$I^{2}$	p value in
							Egger's test
Intake (g d^−1^)							
Dry matter	1551.77 (641.12)	13	31.13 ( - 13.32, 75.59)	0.170	<0.001	86.18	0.811
Organic matter	1494.00 (722.72)	6	45.99 ( - 28.68, 120.66)	0.227	<0.001	95.07	0.871
Crude protein	251.00 (167.38)	6	23.41 (14.47, 32.35)	<0.001	<0.001	92.80	0.571
NDF	587.00 (302.98)	6	22.56 (6.96, 38.16)	0.005	0.024	66.84	0.648
Digestibility (g per kg DM)							
Dry matter	593.99 (200.16)	8	42.35 (22.66, 60.04)	<0.001	<0.001	92.06	0.827
Organic matter	592.13 (200.11)	8	43.79 (26.93, 60.65)	<0.001	<0.001	96.22	0.966
Crude protein	580.80 (207.15)	7	50.17 (29.04, 71.31)	<0.001	<0.001	95.81	0.384
NDF	550.54 (191.85)	8	38.75 (28.31, 49.19)	<0.001	0.012	61.56	0.501
ADF	534.34 (185.45)	8	41.85 (34.40, 49.31)	<0.001	0.492	0	0.846
EE	603.70 (214.75)	7	26.53 (8.97, 44.10)	0.003	<0.001	94.38	0.693
Ruminal fermentation parameters							
pH	5.63 (2.02)	7	0.20 (0.05, 0.36)	0.010	<0.001	82.48	0.079
NH_3_ (mg dL^−1^)	5.35 (5.44)	4	0.16 ( - 0.01, 0.33)	0.064	<0.001	94.57	0.705
Acetate (mol 100 mol^−1^)	59.35 (22.66)	6	- 1.67 ( - 4.41, 1.08)	0.234	<0.001	83.12	0.895
Propionate (mol 100 mol^−1^)	22.19 (10.68)	6	4.28 (3.56, 5.00)	<0.001	0.295	0	0.166
Blood parameters							
Total protein (g dL^−1^)	6.67 (2.25)	8	0.12 ( - 0.02, 0.26)	0.093	0.613	19.09	0.940
Albumin (g dL^−1^)	3.68 (1.41)	4	- 0.11 ( - 0.46, 0.24)	0.524	<0.001	95.13	0.862
Globulin (g dL^−1^)	3.23 (1.32)	5	0.06 ( - 0.04, 0.16)	0.253	0.227	32.77	0.100
Glucose (mg dL^−1^)	65.44 (22.91)	8	8.02 (7.12, 8.92)	<0.001	0.037	43.09	0.087
Antioxidant activity							
Superoxide dismutase (IU mL^−1^)	10.48 (4.56)	5	1.42 ( - 0.13, 2.98)	0.073	<0.001	92.51	0.235
Catalase (IU mL^−1^)	36.74 (45.34)	5	0.21 ( - 0.45, 0.87)	0.536	0.777	0	0.699
Glutathione reductase (IU mL^−1^)	0.07 (0.04)	6	0.01 ( - 0.01, 0.02)	0.363	0.001	95.04	0.152
Glutathione transferase (IU mL^−1^)	0.11 (0.05)	5	0.02 ( - 0.00, 0.03)	0.059	<0.001	91.38	0.099
Glutathione peroxidase (IU mL^−1^)	0.18 (0.08)	5	0.01 ( - 0.01, 0.02)	0.436	0.150	37.04	0.769

N denotes number of comparisons, SD denotes standard deviation, CI denotes confidence interval, and IU denotes international unit.

### 3.2 Blood and antioxidant parameters

Glucose levels were significantly increased by the incorporation of microalgae into goat diets ( p<0.001 ), whereas no significant effect was observed for total protein ( p=0.093 ), albumin ( p=0.524 ), and globulin ( p=0.253 ) (Table 3). Antioxidant activity was not affected by microalgae incorporation for SOD ( p=0.073 ), CAT ( p=0.536 ), GR ( p=0.363 ), G-Tr ( p=0.059 ), or G-Px ( p=0.436 ). Concerning blood parameters, heterogeneity was reported only for albumin ( p<0.001 , $I^{2}=95.13$ ), SOD ( p<0.001 , $I^{2}=92.51$ ), GR ( p<0.001 , $I^{2}=95.04$ ), and G-Tr ( p<0001 , $I^{2}=91.38$ ).

### 3.3 Milk composition and fatty acid profile

The effects of microalgae incorporation on chemical composition and milk FA profile are reported in Table 3. The addition of microalgae into goat diets did not affect milk yield ( p=0.480 ) but increased the yield of lactose ( p<0.001 ), protein ( p<0.001 ), fat ( p<0.001 ), and solid non-fats ( p<0.001 ). Heterogeneity was observed for all milk chemical composition parameters ( p<0.001 , $62.62<I^{2}<100$ ), except for protein yield ( p=0.786 , $I^{2}=0$ ) and lactose ( p=0.885 , $I^{2}=0$ ).

Feeding microalgae to lactating goats affected individual FA and significantly decreased C18:0 ( p=0.001 ) and C18:1 n-9 ( p=0.028 ). This incorporation into goat diets significantly increased C20:5 n-3 ( p=0.027 ) and C22:6 n-3 ( p<0.001 ). This also improved the summaries of FAs, including polyunsaturated FA (PUFA, p=0.039 ) and n-3 ( p=0.006 ). Microalgae incorporation significantly decreased the C14:1 : C14:0 desaturase index ( p= 0.030) and the atherogenicity index (AI; p=0.016 ). Heterogeneity was observed for all FAs ( p<0.001 , $88.50<I^{2}<100$ ).

### 3.4 Meta-regression analysis

**Figure 2:**
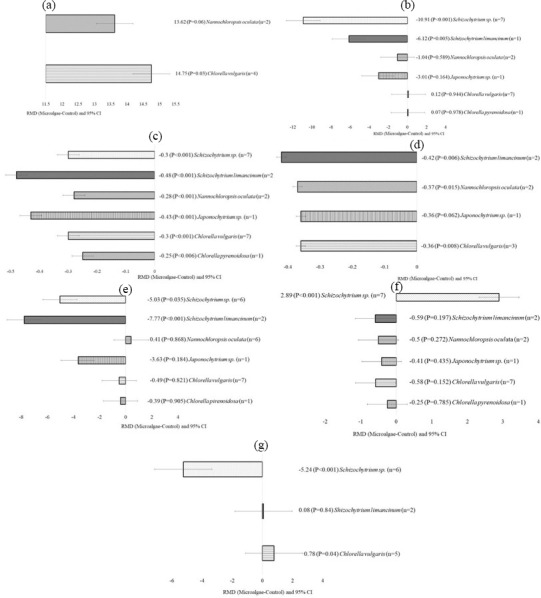
Subgroup analysis (subgroup refers to microalgae species) of microalgae incorporated into dairy goats. RMD denotes raw mean difference between treatment and control diets; n denotes the number of comparisons. **(a)** Fat (g per 100 g FA), **(b)** C18:1 n-9 (g per 100 g FA), **(c)** C18:3 n-3 (g per 100 g FA), **(d)** C20:5 n-3 (g per 100 g FA), **(e)** monounsaturated fatty acids (g per 100 g FA), **(f)** polyunsaturated fatty acids (g per 100 g FA), **(g)** n-6 : n-3.

**Figure 3:**
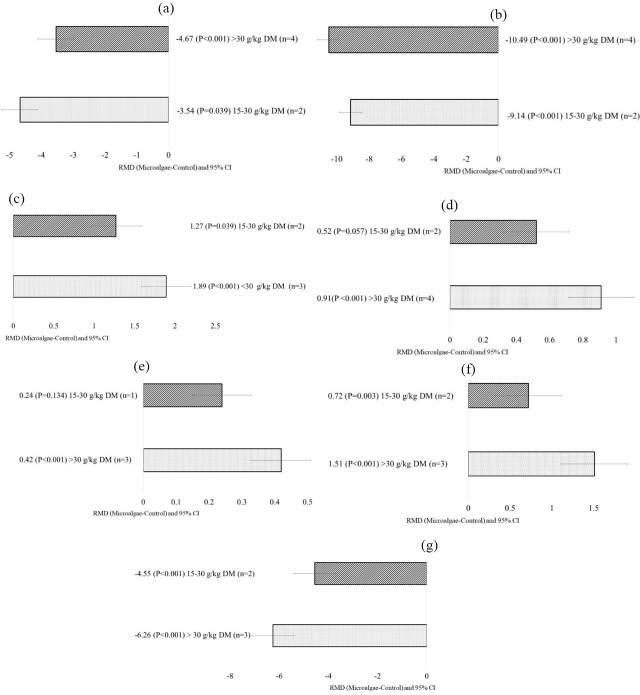
Subgroup analysis (subgroup refers to inclusion level) of microalgae incorporated into dairy goats. RMD denotes raw mean difference between treatment and control diets; n denotes number of comparisons. **(a)** C18:0 (g per 100 g FA), **(b)** C18:1 n-9 (g per 100 g FA), **(c)** C18:2 c9t11 (g per 100 g FA), **(d)** C22:6 n-3 (g per 100 g FA), **(e)** C22:5 n-3 (g per 100 g FA), **(f)** n-3 (g per 100 g FA), **(g)** n-6 : n-3.

**Figure 4:**
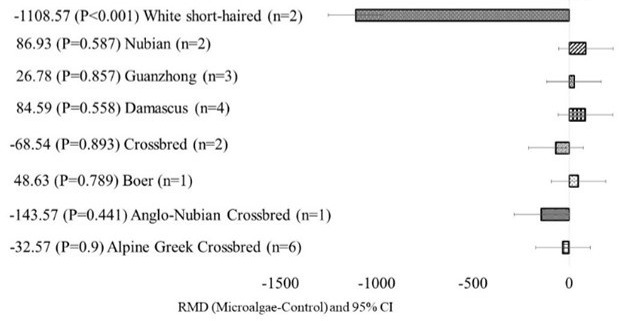
Subgroup analysis (subgroup refers to goat breed) of microalgae incorporated into dairy goats. RMD denotes raw mean difference between treatment and control diets; n denotes number of comparisons. Milk yield in g d^−1^.

As shown in Tables 2–4, the presence of publication bias from Egger's test was not evident for all parameters ( p>0.05 ). Meta-regression analysis was used to investigate the primary contributors to variation among the response variables. The results are shown in Figs. 2, 3, and 4. Among the covariates, animal breed, microalgae species, and inclusion level were the major variables influencing digestibility and fermentation parameters, blood antioxidant activity, milk yield, chemical composition, and FA profile. For response variables in which the covariates accounted for less than 50 % of the heterogeneity, such as DMI (adjusted $R^{2}=47.35$ ), DMD (adjusted $R^{2}=48.71$ ), OMD (adjusted $R^{2}=34.96$ ), total solids (adjusted $R^{2}=19.56$ ), protein (adjusted $R^{2}=34.75$ ), and glutathione reductase (adjusted $R^{2}=7.06$ ), unidentified factors not captured in our meta-analysis might have influenced the impact of microalgae supplementation in goat diets. Thus, these parameters were not included in the subgroup analysis.

**Table 3:** Effects of microalgal supplementation on milk yield, chemical composition, and fatty acid profile of dairy goats.

	Control mean (SD)	N	RMD (95 % CI)		Heterogeneity		Bias
			Random effect	p value	p value	$I^{2}$	p value
Yield (g d^−1^)							
Milk	1564.19 (757.97)	24	- 68.13 ( - 257.04, 120.78)	0.480	<0.001	99.76	0.870
Protein	44.54 (22.68)	8	5.03 (3.74, 6.31)	<0.001	0.786	0	0.975
Lactose	58.23 (42.02)	7	8.98 (7.48, 10.48)	<0.001	0.885	0	0.703
Fat	45.37 (21.82)	10	4.49 (2.36, 6.63)	<0.001	0.009	62.62	0.125
Solid non-fats	84.27 (5.68)	6	14.78 (12.06, 17.49)	<0.001	0.756	0	0.823
Fatty acid profile (g per 100 g FA)							
Stearic (C18:0)	11.44 (4.85)	21	- 1.95 ( - 3.14, - 0.76)	0.001	<0.001	99.24	0.936
Oleic (C18:1 n-9)	18.74 (7.57)	20	- 2.32 ( - 4.56, - 0.08)	0.028	<0.001	99.64	0.232
Linoleic (C18:2 n-6)	3.03 (1.02)	13	- 0.29 ( - 0.58, 0.01)	0.059	<0.001	97.79	0.538
Conjugated linoleic (C18:2 c9t11)	2.07 (0.98)	11	0.45 ( - 0.13, 1.02)	0.126	<0.001	99.61	0.491
α -linolenic (C18:3 n-3)	0.42 (0.39)	21	- 0.01 ( - 0.06, 0.03)	0.578	<0.001	98.06	0.420
Arachidonic (C20:4 n-3)	0.19 (0.08)	9	0.01 ( - 0.03, 0.06)	0.544	<0.001	98.45	0.472
Eicosapentaenoic (C20:5 n-3)	0.11 (0.06)	13	0.03 (0.01, 0.05)	0.027	<0.001	96.98	0.279
Docosahexaenoic (C22:6 n-3)	0.05 (0.05)	12	0.54 (0.24, 0.83)	<0.001	<0.001	100.00	0.351
SFA	70.98 (15.68)	20	- 0.50 ( - 2.01, 1.00)	0.512	<0.001	95.56	0.705
MUFA	25.37 (6.51)	20	- 0.22 ( - 1.75, 1.31)	0.777	<0.001	97.79	0.884
PUFA	3.56 (1.98)	20	0.72 (0.04, 1.40)	0.039	<0.001	99.89	0.196
n-3	0.81 (0.50)	11	0.58 (0.16, 0.99)	0.006	<0.001	99.32	0.345
n-6	3.63 (1.24)	11	- 0.18 ( - 0.48, 0.13)	0.256	<0.001	93.36	0.734
C14:1 : C14:0	0.09 (0.13)	6	- 0.01 ( - 0.02, - 0.00)	0.030	<0.001	90.36	0.050
C16:1 : C16:0	0.03 (0.02)	6	- 0.01 ( - 0.01, 0.00)	0.170	<0.001	88.50	0.897
Atherogenicity index	2.57 (0.99)	10	- 0.24 ( - 0.44, - 0.05)	0.016	<0.001	97.23	0.486

N denotes number of comparisons, SD denotes standard deviation, CI denotes confidence interval, SFA denotes saturated fatty acid MUFA denotes monounsaturated FA, and PUFA denotes polyunsaturated FA.

**Table 4:** Meta-regression of covariate effect on raw mean differences (RMDs) between microalgae and control treatments.

Dependent variable	Meta-regression parameters ( p value)				Adjusted
( Y , RMD)	Intercept	Animal breed	Microalgae	Inclusion level	$R^{2}$ (%)
			species	(g per kg DM)	
Dry matter intake (g d^−1^)	- 90.00 (0.16)	- 90.00 (0.03)	- 200 (0.07)	9.17 (0.62)	47.35
Digestibility (g per kg DM)					
Dry matter	3.00 (0.14)	3.00 (0.14)	48.12 (0.34)	46.94 (0.13)	48.71
Organic matter	- 1.00 (0.09)	- 1.00 (0.21)	54.90 (0.15)	49.21 (0.08)	34.96
Fermentation parameters					
NH_3_ (mg mL^−1^)	0.33 (0.28)	0.33 (0.28)	- 0.01 (0.28)	0.10 (0.19)	76.15
Acetate (mol 100 mol^−1^)	- 2.70 (0.13)	- 2.70 (0.13)	- 2.33 (0.73)	- 1.50 (0.72)	77.48
Blood parameters					
Albumin (g dL^−1^)	- 1.00 (0.01)	- 0.10 (0.01)	- 1.00 (0.01)	–	99.98
Antioxidant activity					
Glutathione reductase (IU mL^−1^)	0.00 (0.61)	0.02 (0.38)	- 0.01 (0.38)	- 0.004 (0.08)	7.06
Superoxide dismutase (IU mL^−1^)	- 0.50 (0.07)	–	- 0.50 (0.50)	0.27 (0.09)	98.64
Yield (g d^−1^)					
Milk	- 144.08 ( <0.001 )	- 150.00 ( <0.001 )	- 62.5 (0.21)	- 71.92 (0.98)	94.19
Fat	4.62 (0.006)	- 5.00 (0.01)	5.70 (0.005)	5.18 (0.002)	85.73
Total solids	0.12 (0.36)	0.12 (0.36)	0.58 (0.09)	–	19.56
Milk composition (%)					
Protein	0.05 (0.16)	0.08 (0.04)	- 0.07 (0.02)	0.08 (0.05)	34.75
Lactose	0.12 (0.04)	0.03 ( <0.001 )	0.09 (0.03)	0.12 (0.003)	58.67
Fat	0.23 ( <0.001 )	- 0.35 ( <0.001 )	0.63 (0.09)	0.11 ( <0.001 )	87.23
Solid non-fats	1.06 (0.01)	0.20 (0.24)	0.26 (0.96)	–	88.13
Fatty acid profile (g per 100 g FA)					
Stearic (C18:0)	2.67 ( <0.001 )	- 3.48 ( <0.001 )	- 0.33 (0.24)	- 0.87 (0.02)	97.42
Oleic (C18:1 n-9)	- 6.83 ( <0.001 )	- 6.39 ( <0.001 )	1.13 (0.006)	0.07 ( <0.001 )	97.11
Conjugated linoleic (C18:2 c9t11)	0.34 ( <0.001 )	0.34 ( <0.001 )	0.03 (0.16)	0.10 (0.005)	98.98
α -linolenic (C18:3 n-3)	0.18 ( <0.001 )	- 0.17 (0.26)	0.29 ( <0.001 )	- 0.01 (0.88)	98.91
Eicosapentaenoic (C20:5 n-3)	0.01 (0.15)	0.05 (0.52)	0.04 (0.01)	0.02 (0.007)	78.96
Docosapentaenoic (C22:5 n-3)	0.28 (0.03)	0.02 (0.03)	0.02 (0.12)	- 0.001 (0.007)	90.29
Docosahexaenoic (C22:6 n-3)	- 0.10 (0.37)	0.49 (0.02)	0.02 (0.12)	0.21 (0.01)	46.67
SFA	- 6.55 (0.05)	3.62 (0.38)	- 3.15 (0.01)	- 0.60 (0.99)	66.81
MUFA	4.56 (0.02)	- 4.52 (0.08)	2.09 (0.01)	0.39 (0.25)	77.65
PUFA	0.69 ( <0.001 )	0.05 ( <0.001 )	0.60 (0.23)	0.10 ( <0.001 )	99.59
n-3	0.07 (0.33)	- 0.02 (0.01)	0.33 (0.14)	0.16 (0.01)	52.68
n-6 : n-3	0.15 (0.002)	- 0.18 ( <0.001 )	- 0.65 ( <0.001 )	- 0.90 (0.003)	92.09

Regarding the effects of the animal breed covariate, microalgae incorporation into goat diets reduced milk yield for the White Short-Haired breed ( p<0.001 ), whereas no change was observed in the other animal breeds. Total solids increased when microalgae were introduced into Boer ( p<0.001 ) and Damascus ( p=0.005 ) diets and did not change for the other breeds. Milk fat significantly increased in the native Hungarian breed ( p=0.019 ) and decreased in Alpine–Greek crossbreeds ( p=0.013 ). Milk from Damascus goats showed significant decreases in C18:2 c9t11 ( p=0.006 ) and increased C18:0 ( p=0.023 ), C18:1 n-9 ( p<0.001 ), C18:3 n-3 ( p=0.010 ), C22:5 n-3 ( p<0.001 ), and C22:6 n-3 ( p=0.009 ) when supplemented with microalgae. When microalgae was incorporated into the Alpine–Greek crossbreed diet, there was a significant decrease in C18:0 ( p=0.003 ) and C18:1 n-9 ( p<0.001 ) and a significant increase in C18:2 c9t11 ( p<0.001 ), C18:3 n-3 ( p=0.010 ), C22:5 n-3 ( p<0.001 ), C22:6 n-3 ( p=0.009 ), PUFA ( p<0.001 ), and n-3 ( p<0.001 ). For the Boer breed, microalgae incorporation increased C18:0 ( p=0.031 ) and C18:1 n-9 ( p<0.001 ) and decreased C18:2 c9t11 ( p<0.001 ). Nubian goats showed significant increases in C18:0 ( p=0.007 ), C18:1 n-9 ( p<0.001 ), C18:3 n-3 ( p=0.016 ), and MUFA ( p=0.020 ) and a significant decrease in C18:2 c9t11 ( p<0.001 ). Native Hungarians showed increased C18:1 n-9 ( p<0.001 ) and C18:3 n-3 ( p=0.003 ).

Microalgae species also differ in terms of their effects on goat milk and performance. Incorporation of *Schizochytrium* sp. into goat diets significantly decreased milk C18:0 ( p=0.002 ), C18:1 n-9 ( p<0.001 ), C18:3 n-3 ( p<0.001 ), and MUFA ( p=0.035 ) and significantly increased C20:5 n-3 ( p=0.012 ), C22:6 n-3 ( p=0.014 ), and PUFA ( p<0.001 ). The incorporation of *Japonochytrium* sp. decreased milk yield ( p=0.026 ) and C18:3 n-3 content ( p<0.001 ). When *Chlorella vulgaris* was incorporated into goat diets, an increase in TS ( p=0.018 ) and a decrease in C18:2 c9t11 ( p=0.010 ), C18:3 n-3 ( p<0.001 ), and C22:5 n-3 ( p=0.008 ) were observed. The incorporation of *Schizochytrium limacum* increased SFA ( p=0.003 ) and decreased C18:1 n-9 ( p=0.005 ), C18:3 n-3 ( p<0.001 ), C20:5 n-3 ( p=0.013 ), C22:5 n-3 ( p=0.006 ), and MUFA ( p=0.010 ). When *Nannochloropsis oculata* was incorporated into goat diets, a decrease in C18:3 n-3 ( p<0.001 ) and C22:5 n-3 ( p=0.015 ) was observed.

The quality and production of goat milk were also affected by the level of microalgae incorporation. Higher levels of microalgae incorporation ( >30 g per kg DM) significantly increased C18:2 c9t11 ( p<0.001 ), C20:5 n-3 ( p=0.001 ), C22:5 n-3 ( p<0.001 ), C22:6 n-3 ( p<0.001 ), PUFA ( p<0.001 ), and n-3 ( p<0.001 ) and significantly decreased C18:0 ( p<0.001 ) and C18:1 n-9 ( p<0.001 ). Moderate incorporation of 15–30 g per kg DM microalgae increased C18:2 c9t11 ( p=0.039 ), PUFA ( p<0.001 ), and n-3 ( p=0.003 ) and decreased C18:0 ( p=0.039 ) and C18:1 n-9 ( p<0.001 ).

## 4 Discussion

### 4.1 Overview of studies used in the analysis 

The findings from this meta-analysis indicate that the majority of studies will be conducted from 2015 to 2022. This trend might be associated with the global initiative urging researchers to explore alternative natural feed supplements to enhance livestock productivity. This research spanned eight countries, representing four continents, which shows that research on microalgae as animal feed is emerging worldwide.

### 4.2 Microalgae overall effect

Ruminants have been fed microalgae as an alternative, low-cost, and highly digestible feed (Niccolai et al., 2019). The first expected barrier to feeding large quantities of microalgae to ruminants is their palatability problems due to vegetable, grass, and cucumber flavors (Van Durme et al., 2013). In a subsequent study, DM intake was not affected by microalgae incorporation into goat diets, while a meta-analysis of small ruminants receiving microalgae showed an improved intake (Orzuna-Orzuna et al., 2023). As expected, microalgae improved the digestibility of DM, OM, CP, EE, and fibers (NDF and ADF). The improved nutrient digestibility indicates heightened rumen microflora activity following microalgal supplementation. Microalgae possess approximately 67 % UFA in terms of their overall total FA content (Maltsev and Maltseva, 2021), and supplementation with UFA may have decreased the number of protozoa known to engulf bacterial cells. Boeckaert et al. (2007) observed that microalgae with high levels of UFA reduced the populations of certain ciliates, including *Isotricha intestinalis*, *Isotricha prostoma*, and *Epidinium caudatum*. The decrease in the number of protozoa is linked to reduced predation of the bacterial population and improved digestibility (Kholif and Olafadehan, 2021).

For fermentation parameters, microalgae supplementation in goat diets did not affect ruminal acetate, which might indicate that the levels of UFA present in the microalgae in the current experiment were adequate to improve the performance of goats without affecting ruminal cellulolytic bacterial activity. Moreover, microalgal supplementation of goats increased ruminal pH and propionate levels (Fig. 2). Propionate is considered to be the primary gluconeogenic volatile FA used for lactose and glucose biosynthesis (Wang et al., 2023), which explains the improvement in serum glucose concentrations. Approximately 73 % of the glucose produced in the livers of ruminants originates from ruminal propionic acid (Oh et al., 2015). The incorporation of microalgae into ruminant diets aims to improve milk yield and quality. Contrarily to our hypothesis, the meta-analysis conducted herein outlined that microalgae changed milk quality and composition but not yield. Although microalgal diets are rich in polyunsaturated fatty acids, milk fat depression (MFD) is avoided, and milk fat is improved.

Regarding the phytochemical composition of microalgae, several studies have reported the presence of molecules such as polysaccharides, sterols, vitamins, and pigments (e.g., carotenoids), which have antioxidant roles (Coulombier et al., 2021). However, no significant effect was observed in this meta-analysis. Unexpectedly, in a study conducted on the same species aiming to enrich goat products with PUFA via microalgae supplementation, high dietary levels of *Schizochytrium* sp. (40 and 60 g d^−^) increased nicotinamide adenine dinucleotide phosphate (NADPH) oxidase activity in blood plasma, resulting in superoxide anion formation and pro-oxidation, whereas no effect was observed with lower supplementation (20 g). The following observation emphasizes the importance of using the correct dosage, which is highlighted by subgroup analysis. In a study conducted on sheep, Christodoulou et al. (2023) reported that *Spirulina* dietary supplementation significantly increased the antioxidant activity of animals through higher activities of superoxide dismutase, glutathione peroxidase, and catalase.

Microalgae are sources of PUFA that are reported to inhibit rumen biohydrogenation and, as a result, increase CLA and C18:1 trans-11 substrates (Boukrouh et al., 2023a). These latter FAs can also increase through the activity of Δ 9 desaturase in the mammary gland. As milk C14:0 is synthesized de novo in the udder and because milk C14:1 originates exclusively from C14:0 desaturation at this site (Lock and Garnsworthy, 2003), the Δ 9 : C14 ratio is considered to be the marker for Δ 9 desaturase activity, which was decreased in the following study. In a study comparing fish oil and microalgae effects on goat milk quality, no significant effect was observed for goats receiving microalgae, while there was an improved effect for goats receiving fish oil (Beyzi and Dallı, 2023).

### 4.3 Microalgae species

The response to microalgae incorporation differed according to microalgae species. In the case of *Chlorella vulgaris*, despite its higher concentration in C18:2 n-6, isomerization of C18:2 n-6 to CLA was inhibited when it was incorporated into goat diets, which explains the decrease in CLA in milk (Tsiplakou et al., 2018). The reason for the increased C22:6 n-3 and PUFA with the incorporation of *Schizochytrium* sp. into goat diets compared to other microalgae species is probably the higher concentration in these FAs and the inhibition of ruminal biohydrogenation (Półbrat et al., 2021). A previous study has reported that the DHA content of *Schizochytrium* sp. accounts for 35 % of the total FA content (Adarme-Vega et al., 2012). Moreover, the higher concentration of PUFA compared to *Chlorella vulgaris* could be the reason for the reduced fat content. These PUFA are known to modulate rumen biohydrogenation, leading to the accumulation of biohydrogenation intermediates such as trans-10, cis-12 conjugated linoleic acid, which are potential inhibitors of de novo milk fat synthesis, leading to MFD (Baumgard et al., 2001). The improvement in C18:3 n-3 and C22:5 n-3 content when *Nannochloropsis oculata* was fed to goats could be due to its higher content of n-3 FA, DHA, and EPA (3.2 % and 21.5 % fat, respectively) (Durmic et al., 2014). In addition, C18:3 n-3 is the precursor of n-3 FAs such as EPA and DHA, which are essential for many human metabolic processes and serve as preventive measures against coronary heart disease (Bhat et al., 2023).

### 4.4 Supplementation level

The level of microalgae incorporation into goat diets affects animal production and performance. Some studies have suggested that supplementation with low doses of microalgae decreases DNA damage and enhances the blood oxidative status in goats and sheep (Vignaud et al., 2023). We expected improvements in general health and increased production. Moderate incorporation of microalgae (15–30 g per kg DM) improved only CLA, PUFA, and n-3 compared to higher incorporation ( >30 g per kg DM). However, even moderate incorporation can be beneficial to health. An improvement in n-3 FA can lower human blood triglycerides, reduce the risk factors for coronary heart disease, and minimize the likelihood of thrombosis that leads to heart attack (Yagi et al., 2017).

Stearic acid (C18:0) is the ultimate product of the ruminal biohydrogenation process (Dewanckele et al., 2020). The decrease in stearic acid in the milk of goats receiving both medium (15–30 g per kg DM) and high levels of microalgae ( >30 g per kg DM) suggests that the biohydrogenation process is sensitive even to moderate doses of microalgae incorporation. The lower biohydrogenation of PUFA reduced the availability of C18:0 in the mammary gland, which led to the termination of de novo synthesis of C18:1 n-9 through Δ9 desaturase activity (Dewanckele et al., 2020). CLA was raised in the milk of goats that received medium- and high-microalgae diets as a result of the partial inhibition of dietary PUFA biohydrogenation.

### 4.5 Animal breeds

The impact of animal breed on the goat reaction to microalgae supplementation may be due to differences in rumen microbial communities among goat breeds (Shi et al., 2008). According to Morand-Fehr et al. (2007), variations in milk yield among different goat breeds across various countries can be attributed to differences in genetic and environmental factors, as well as the type of management systems implemented in these regions. Shamay et al. (2000) suggested that, under stressful environmental conditions, the milk composition of goats remains unaffected if they are well-adapted to their environment.

In a study comparing the milk composition of different goat breeds, the lowest fat content was registered for the Alpine breed compared to Damascus and Boer breeds (Ferro et al., 2017), which could explain the decreased milk fat when microalgae were supplemented in the diets of Alpine–Greek crossbreeds as the potential of crossbreeds could not exceed that of Damascus and Boer (Ferro et al., 2017). When selecting for higher milk yield, the concentration of milk fat could decrease due to dilution, which could also be the reason for the decreased milk fat for Alpine–Greek crossbreeds (Goetsch et al., 2011). However, Alpine–Greek crossbreeds showed increased milk C18:3 n-3, CLA, DPA, DHA, PUFA, and n-3 when animals were fed microalgae compared to other breeds, which could be due to the concentration of efforts to enhance the milk quality of the hybrid. It is noteworthy that certain breeds (e.g., White Short-Haired, Boer) and microalgae species have been represented by few studies (often just one), which underscores the necessity for additional research to thoroughly investigate these interactions. Although the meta-analysis employed a random-effects model to account for heterogeneity, the validity of the conclusions drawn for underrepresented subgroups remains limited, and the variability emphasizes the need for more studies to comprehensively explore these interactions. Further research should address these gaps by including a broader range of breeds, experimental conditions, and microalgae species to strengthen the evidence base and to provide more conclusive insights into the role of microalgae supplementation in goat nutrition.

### 4.6 Investigation of heterogeneity and publication bias

Heterogeneity in meta-analysis is a test used to detect the difference between studies, and high heterogeneity values ( $I^{2}>50$ %) observed in our analysis reflect the expected variability between studies, implying different incorporation methods in goats. The heterogeneity was also highlighted by the higher SD for some variables and probably arises from variability in factors such as diet composition, microalgae species, experimental design and duration, and animal breed. To account for this, we employed a random-effects model (Langan, 2022), which provides a robust estimate in the presence of heterogeneity, assuming that the true effects vary across studies.

Another important parameter in meta-analyses is publication bias, which is generally caused by the orientation of reviewers and journal editors to favor the publication of studies showing significantly positive results rather than those reporting negative findings (Nosek et al., 2022). In the present meta-analysis, despite the high heterogeneity, the absence of significant publication bias suggests that the variability is probably due to natural differences between studies rather than biased reporting of the results, which strengthens the reliability of the overall conclusions. Meta-regression and subgroup analyses were used as a result of high heterogeneity to identify sources of variation and to better contextualize the findings. The employed moderators, such as microalgae species, inclusion levels, and animal breeds, provided a deeper understanding of the conditions where microalgae supplementation is most beneficial.

## 5 Conclusions

Research on microalgae as an alternative feed for goats has recently emerged. This meta-analysis provides fundamental information concerning the potential effects of microalgal supplementation on goat milk production and quality. The results showed that microalgae supplementation in goat diets did not affect dry matter intake or milk production, but the FA profile of milk was improved. Our findings also suggest that supplementation with >30 g per kg DM is necessary to improve the milk FA profile and to produce healthier goat milk. The best FA profile was obtained with the use of *Schizochytrium* sp. and when microalgae were incorporated into the Alpine–Greek crossbreed. The results revealed the presence of heterogeneity but the absence of publication bias, and meta-regression analysis illustrated that covariates explained some of the sources of variation. The aforementioned results may serve as a valuable reference for animal nutritionists and policymakers, helping them make evidence-based decisions concerning the utilization of microalgae feed supplements to enhance goat performance.

## Data Availability

The datasets presented in this study are available upon request from the corresponding author.

## Code availability

The software code presented in this study is available upon request from the corresponding author.
